# FK506 Induces the TGF-β1/Smad 3 Pathway Independently of Calcineurin Inhibition to Prevent Intervertebral Disk Degeneration

**DOI:** 10.3389/fcell.2020.608308

**Published:** 2020-12-10

**Authors:** Jun Ge, Yingjie Wang, Qi Yan, Cenhao Wu, Hao Yu, Huilin Yang, Jun Zou

**Affiliations:** Department of Orthopaedic Surgery, The First Affiliated Hospital of Soochow University, Suzhou, China

**Keywords:** FK506, disk degeneration, TGFβ, Smad3, signal pathway

## Abstract

**Background:**

Intervertebral disk (IVD) degeneration is the most common cause of lower back pain. Inhibiting inflammation is a key strategy for delaying IVD degeneration. Tacrolimus (FK506) is a potent immunosuppressive agent that is also beneficial to chondrocytes via alleviating inflammation. However, the potential function of FK506 in IVD and the underlying mechanisms remain unknown. The current study is aim at exploring the underlying mechanism of FK506 in preventing IVD degeneration.

**Methods:**

Cell morphology was imaged using an optical microscope. mRNA levels of nucleus pulposus (NP) matrix components were determined by qRT-PCR, and protein expression NP matrix components was assessed by western blotting. A rat caudal IVD degeneration model was established to test for FK506 *in vivo*.

**Results:**

FK506 improved the morphology of NP cells and the cell function at both the mRNA and protein level. FK506 could attenuate NP degeneration induced by IL-1β. Furthermore, FK506 exerted its function via TGFβ/Smad3 activation instead of through calcineurin inhibition. Inhibition of the TGF-β pathway prevented the protective effect of FK506 on IVD degeneration. In an *in vivo* study, FK506 injection reversed the development of rat caudal IVD degeneration influenced by Smad3.

**Conclusion:**

Our current study demonstrates the positive effect of FK506 on delaying the degeneration of IVD via the TGFβ/Smad3 pathway.

## Introduction

Lower back pain has become a major difficulty in the aging population worldwide (Clark and Horton, 2018), and a significant cause of lower back pain is intervertebral disk (IVD) degeneration (Freemont, 2009). Recent studies have shown elevated expression of multiple inflammatory mediators in degenerated intervertebral disk tissue, suggesting a plausible role for inflammatory mediators in the process of IVD degeneration (Johnson et al., 2015; Xu et al., 2016; Zhao et al., 2017). Inhibiting inflammation is a key strategy for delaying or even reversing IVD degeneration (Peng et al., 2006).

Tacrolimus (FK506) is a potent immunosuppressive agent that is widely used for treating immunological disorders. FK506 exerts its therapeutic effects via suppression of T-cell activation (Karagun et al., 2017). The inhibition of calcium/calmodulin-dependent calcineurin (Cn) represents a classical pathway involving FK506 in immunosuppression, and this inhibition results in inhibition of Cn mediated dephosphorylation of transcription factors within the nuclear factor of activated T-cells (NFATs) family.

According to the results from numerous immunological preparations, FK506 does not markedly affect bone marrow cell differentiation and proliferation; however, FK506 has elicited broad interest within the fields of orthopedics and sports medicine (Sakuma et al., 2010). A series of clinical trials and animal experiments (Yocum et al., 2003; Miyata et al., 2005; Furst et al., 2010) have shown that FK506 could significantly alleviate inflammatory reactions and relieve symptoms of rheumatoid arthritis. A small number of recent studies have also revealed that FK506 may induce chondrogenic differentiation of murine chondroprogenitor cells (Nishigaki et al., 2002; Nakamura et al., 2009). Moreover, FK506 could protect articular cartilage by reducing the degradation of the extracellular collagen matrix (Siebelt et al., 2014). Therefore, FK506 possesses the potential to treat degenerative joint diseases or non-inflammatory arthritis. Considering that the normal nucleus pulposus cells (NPCs) exhibit similar phenotype to chondrocytes, we hypothesized that FK506 may exert a therapeutic effect on the NPCs in the context of IVD degeneration.

Despite the current knowledge regarding FK506, the exact mechanism by which FK506 protects the articular cartilage and alleviates arthritis remain unidentified. Several studies have reported that the Cn/NFAT signaling cascade (the classical FK506 pathway) plays a vital role in bone remodeling and chondrogenesis.

*In vivo* and *in vitro* data (Wang et al., 2009, 2014; Greenblatt et al., 2013; Zhang et al., 2016) from both mice and humans provide a compelling evidence for a crucial role for NFAT family members in maintaining cartilage and joint health. These results suggest that NFAT1 is an essential transcriptional regulator of chondrocyte homeostasis in adult articular cartilage. However, several earlier *in vitro* studies suggest that NFAT signaling can act as a negative regulator in chondrocyte differentiation, as this signaling induces catabolic genes (e.g., ADAMTS 4 and 9) (Thirunavukkarasu et al., 2006; Yaykasli et al., 2009; Kim et al., 2014). Based on these disparate findings, we further hypothesize that, even as a calcineurin/NFAT inhibitor, FK506 may exert its function in chondrocytes via a calcineurin-independent pathway. In regard to the effect of FK506 on IVD degeneration, the involvement of the calcineurin-independent pathway in intervertebral disk degeneration has not been examined.

In our previous study, we demonstrated that activation of the TGF-β/SMAD pathway could effect the inhibition of the inflammatory response, and the delayed degeneration, and protection of the intervertebral disk (Yang et al., 2016). Giordano et al. (2008) and Lan et al. (2015) reported that FK506 could activate the TGF-β pathway. Based on these findings, we speculated that FK506 may delay the degeneration of the intervertebral disk through the TGF-β/Smad 3 pathway. The objective of the current work is to confirm the therapeutic efficacy of FK506 in delaying IVD degeneration and to explore the underlying mechanisms.

## Materials and Methods

### Materials

Interleukin-1 beta (IL-1β, R&D Systems, Minneapolis, MN, United States) was dissolved in sterile phosphate buffer saline (PBS) containing 0.1% bovine serum albumin (BSA) at a concentration of 25 μg/ml and then diluted to different concentrations within culture medium prior to cell exposure. FK506 (Prograf, Astellas Ireland Co., Ltd, Killorglin, Co. Kerry, Ireland) was diluted to different concentrations within culture medium prior to cell exposure.

### Isolation and Culture of Primary Nucleus Pulposus Cells

The method of isolation of primary NPCs was consistent with our previous protocol (Ge et al., 2020b). Briefly, a 8-week-old clean (GB 14922-1994) male Sprague Dawley (SD) rat weighing 225–270 g was mercy sacrificed. After routine disinfection, the tail was cut from the root and delicate separated. The nucleus pulposus (NP) tissue was harvested and placed into PBS containing 100x penicillin-streptomycin. After digestion with Type II collagenase solution (0.2%), the digestion process was neutralized with DMEM/F12 complete culture medium. Then, the solution was centrifuged and the supernatant was aspirated. NPCs were then collected and resuspended. Ten intervertebral disks are necessary for the isolation primary NPCs at one time. New primary NPCs were isolated and subcultured to the 3rd generation before each experiment (including repeated experiments). The method of culture of primary NPCs was consistent with our previous protocol (Ge et al., 2020a). NPC were cultured in DMEM medium supplemented with 10% (v/v) fetal bovine serum, 100 U/ml penicillin, 100 mg/ml streptomycin, and 1% NPCs Growth Supplement (ScienCell, Carlsbad, CA, United States) at 37°C and 5% CO2. For qRT-PCR and western blotting assays, the cells were seeded into the 10 cm dish, when the cell confluency reached 80%, different concentrations of IL-1β and FK506 was added, and the cells were cultured for 24 h.

#### qRT-PCR

qRT-PCR was performed in accordance with our previous protocol (Ge et al., 2020a). Total RNA from cells was extracted using the RNeasy kit (QIAGEN, Hilden, Germany). The cDNA was synthesized with a RevertAid First Strand cDNA Synthesis Kit (Thermo Scientific, Tewksbury, MA, United States) according to the manufacturer’s protocol. Quantitative real-time PCR was performed using an ABI 7500 sequence detection system (Applied Biosystems, Foster City, CA, United States) and Power SYBR Green PCR Master Mix (Applied Biosystems, Foster City, CA, United States). Primer sequence of each target gene has been listed in Table 1. Each target probe was amplified in a separate 96-well plate. All samples were incubated at 95°C for 10 min. They were then cycled for 40 cycles as follow: 95°C for 15 s and 60°C for 1 min. The results were captured and evaluated by use of SDS software (Applied Biosystems, Foster City, CA, United States). For each sample, the relative amount of the target mRNA was determined and normalized to *GAPDH*. The experiment was independently repeated three times.

**TABLE 1:** Primer sequence.

COLII-F	TGCTGCCCAGATGGCTGGAGGA
COLII-R	TGCCTTGAAATCCTTGAGGCCC
Smad2-F	GACACCAGTTTTGCCTCCAGTAT
Smad2-R	CAGAGGCGGAAGTTCTGTTAGG
Smad3-F	GGCCACCGTCTGCAAGAT
Smad3-R	GCGAACTCCTGGTTGTTGAAG
SOX9-F	AGGTGCTCAAAGGCTACGAC
SOX9-R	GTAATCCGGGTGGTCCTTCT
ACAN-F	CTACCGCTGCGAGGTGATG
ACAN-R	AGTCGAGGGTGTAGCGTGTAGAG
COLX-F	TGCTGCCACAAATACCCTTT
COLX-R	GTGGACCAGGAGTACCTTGC

#### Western Blotting

Western blotting was performed according to our previous protocol (Ge et al., 2020a). The total protein of cells was extracted using RIPA buffer (50 mM Tris, pH 7.4; 150 mM NaCl; 1% NP-40; 0.5% sodium deoxycholate). The solution was then collected and centrifuged at 13000 *g* at 4°C for 20 min. The supernatant was collected, and the protein concentrations were quantified using the BCA Kit (Beyotime, China). A 5x loading buffer (250 mM Tris-HCl, pH6.8; 10% [W/V], SDS; 0.5% [W/V] black-pigmented Bacteroides; 50% [V/V] glycerinum; 5% [W/V] β-mercaptoethanol) was added to each sample and subsequently boiled, and 40 μg of protein was separated on 10% sodium dodecyl sulfate-polyacrylamide gels (SDS-PAGE) under reducing conditions and then transferred on to PVDF membranes. After blocking non-specific binding sites with 5% low-fat milk TBS solution, PVDF membranes were incubated with primary antibodies (anti-Sox9, anti-collagen II, anti-aggrecan, Abcam, Cambridge, MA, United States; anti-Smad3, anti-Smad2, anti-p-Smad3, and anti-p-Smad2, Abclonal, Woburn, MA, United States) in 2.5% low-fat milk TBS solution overnight at 4°C. Horseradish peroxidase-conjugated goat anti-rabbit antibodies (Cell Signaling Technology, Danvers, MA, United States) diluted 1:10,000 were used as secondary antibodies. Then, immunoreactive proteins were visualized using a chemiluminescence kit (Thermo Scientific, Tewksbury, MA, United States) followed by exposure under a gel imaging device (Fujifilm, Japan). The images were analyzed with ImageJ software. The experiment was independently repeated three times. The relative protein expression level was compared with the control group.

### Establishment of Rat Caudal IVD Models

The method for the establishment of rat caudal IVD models was consistent with our previous protocol (Ge et al., 2020b). A total of 18 clean SD rats (male, weighing 400 ± 20 g) were carefully tested by X-ray and magnetic resonance before the experiment to check for congenital malformations or disk degeneration. Then, the rats were randomly divided into three groups of six rats each using the digital table method, including saline group, CAPI group, and FK506 groups. The rats were anesthetized by intraperitoneal injection of ketamine hydrochloride (50 mg/kg) and xylazine hydrochloride (5 mg/kg). Co 8/9 vertebral gap was located according to preoperative X-rays. Then the rat was fixed onto the operating table, and 18G injection needles were selected, according to our previous study (Qian et al., 2019), to pierce the Co 8/9 vertebral gaps. The needle tips were inserted and complete penetrated to the opposite side perpendicularly to the rat tail. The tips were then rotated 360° and retracted after 30 s. Each group was then treated with the corresponding drugs. Saline, CAPI (20 μg/ml), and FK506 (20 μg/ml), respectively, were injected at 2 μl into the degenerated disks using a 26G needle, according to our previous study (Qian et al., 2019).

After the operation, rats were allowed free movement in the cage, free to water and food. They were closely observed for the presence of urinary retention and infection.

### MRI Examination

MRI examination was performed in accordance with our previous protocol (Ge et al., 2020b). Rats were anesthetized and then scanned by MRI at 1, 2, and 4 weeks after the operation. Intervertebral disk signals were obtained on a 1.5T Magnetic Resonance (MR) scanner (Philips Eclipse) using the following parameters of T2-weighted sagittal plane: TR/TE: 3500/102 ms, FOV: 15.0, thickness: 3 mm, and interval: 0 mm. The degree of disk degeneration was assessed according to signal intensity on a T2-weight image (T2WI) of the intervertebral disk. The degree of disk degeneration was assessed by signal intensity on T2-weight image (T2WI) of the intervertebral disk, and graded with a five-grade Pfirrmann system (Pfirrmann et al., 2001) (Table 2). Grading is performed on T2-weighted midsagittal (repetition time 5000 ms/echo time 130 ms) fast spin-echo images. The grading was performed independently by two experienced spinal surgeons. When the grades diverged, a third and more senior spinal surgeon was asked to evaluate the results, and three spinal surgeons negotiated and gave the final decision.

**TABLE 2:** Pfirrmann system.

Grade	Pfirrmann System			
	Structure	Signal	Disc Height	Distinction between nucleus and annulus
I	Homogeneous	Bright hyperintense white signal intensity	Normal	Clear
II	Inhomogeneous	Hyperintense white signal	Normal	Clear, with or without horizontal gray bands
III	Inhomogeneous	Intermediate gray signal intensity	Normal or slightly decreased	Unclear
IV	Inhomogeneous	Hypointense dark gray signal intensity	Normal or moderately decreased	Lost
V	Inhomogeneous	Hypointense black signal intensity	Collapsed	Lost

### Histological Examination

Histological examination was performed according to our previous protocol (Ge et al., 2020b). After the imaging examination was completed, the rats were mercy sacrificed. The Co 8/9 disks were harvested and fixed with 10% neutral formaldehyde at room temperature for 1 day. The tissues were decalcified in 20% EDTA for 2 weeks and embed into paraffin blocks. The blocks with samples were sliced into horizontal sections. Slices were dewaxed in xylene twice for 5 min, dehydrated in graded ethanol, and stained with hematoxylin and eosin. The morphology of the intervertebral disk was observed and scored under a light microscope according to our previously published methods (Table 3). The scoring was performed independently by two experienced spinal surgeons. When the scores diverged, a third and more senior spinal surgeon was asked to evaluate the results, and three spinal surgeons negotiated and gave the final decision.

**TABLE 3:** Grading for morphology.

		Morphology Change Under Optical Microscope	Grade
I	Annulus Fibrosus (AF)	Normal texture and free of damage and distortion	1
		The damaged and distortion area is less than 30%	2
		The damaged and distortion area is more than 30%	3
II	Boundary between AF and NP	Normal	1
		Micro disrupted	2
		Medium or severe disrupted	3
III	NP Cells	Normal cells with large amounts of vacuoles	1
		Cells and vacuoles decreased slightly	2
		Cells decreased moderately or severely without vacuoles	3
IV	NP Matrix	Normal gel appearance	1
		Slightly congealed	2
		Moderate or severe condensation	3

### Immunohistochemical Staining of Collagen II and SMAD3

The expression of collagen II (Abcam, Cambridge, MA, United States), collagen X (Abcam, Cambridge, MA, United States), and Smad3 (Cell Signaling Technology, Danvers, MA, United States) was detected using immunohistochemistry. The slides were dewaxed, dehydrated, and incubated in 3% H_2_O_2_ at 37°C for 10 min. The slides were then washed and boiled in 0.01M citric acid buffer for 20 min. Next, they were blocked in goat serum at 37°C for 10 min. Slides were then incubated with primary antibody at 4°C overnight and then in secondary antibody (Bioworld, Dublin, OH, United States) at 37°C for 30 min. Slides were finally counterstained with hematoxylin and subsequently evaluated with Image Pro Plus 6.0.

### Statistical Analysis

All quantitative data are presented as mean ± SD. Statistical analyses were performed using one-way ANOVA followed by Dunnett’s multiple comparisons. For non-parametric data, the Kruskal-Wallis test was performed. Differences with values of *p* < 0.05 were considered statistically significant.

## Results

### FK506 Can Improve the Function of Nucleus Pulposus Cells

**FIGURE 1:**
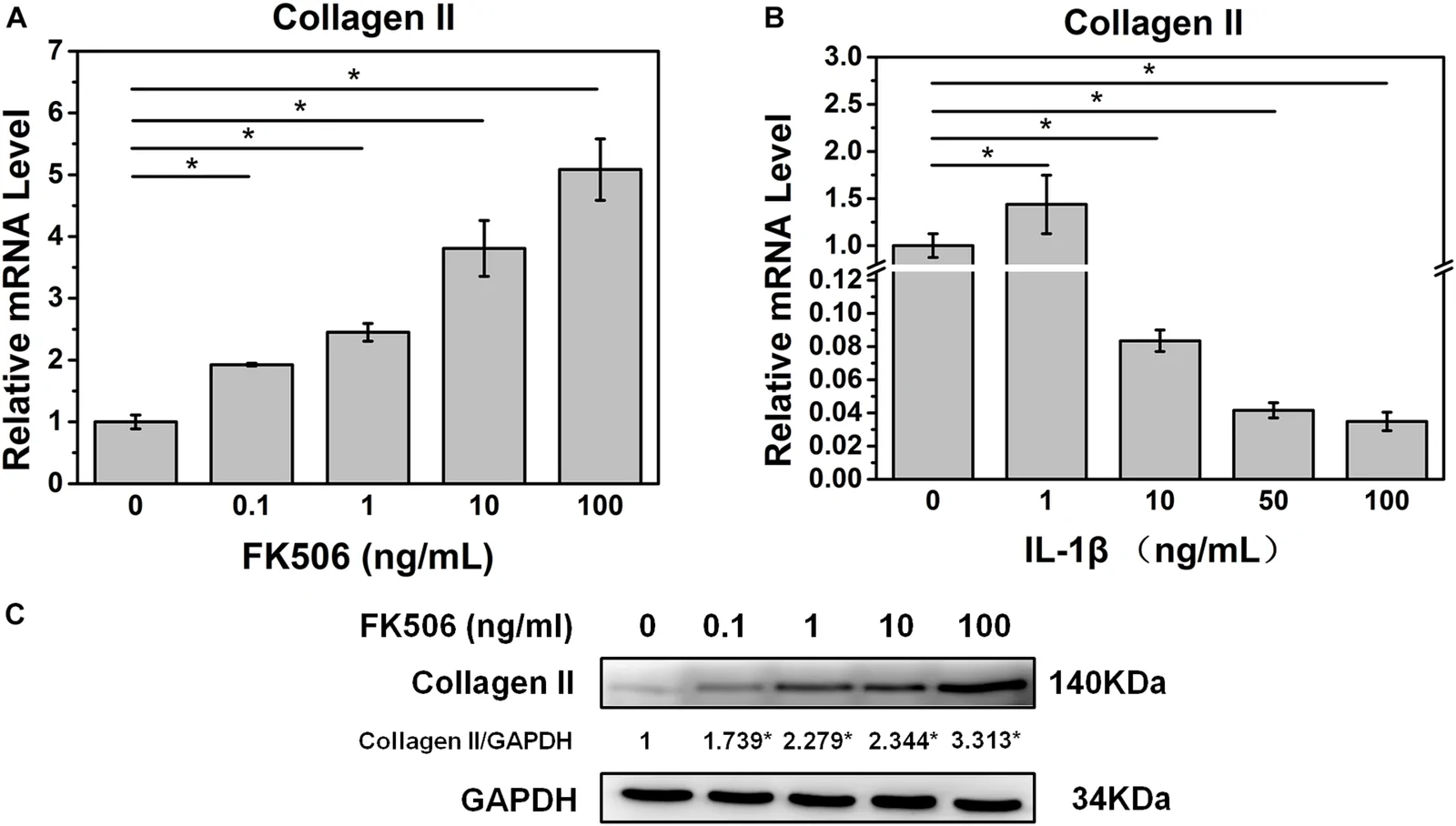
FK506 improves the function of NPCs, and 10 ng/ml IL-1β treatment can induce degeneration of NPCs. **(A)** The mRNA level of type II collagen increased as the concentration of FK506 increased. **(B)** When IL-1β concentration is higher than 10 ng/mL, the mRNA level of type II collagen is significantly reduced (*p* < 0.05). **(C)** The protein level of type II collagen increased as the level of FK506 increased (*p < 0.05).

Initially, we investigated the effect of FK506 on the behavior of NPCs. qRT-PCR revealed that FK506 could increase the level of collagen II in a dose-dependent manner (Figure 1A). With respect to protein expression, western blot analysis yielded similar results indicating that FK506 could increase the gene expression of collagen II in a dose-dependent manner (Figure 1C). Thus, FK506 could improve the levels of NP-related proteins and attenuate the degeneration of NPCs.

### FK506 Can Attenuate Nucleus Pulposus Degeneration Induced by IL-1β

**FIGURE 2:**
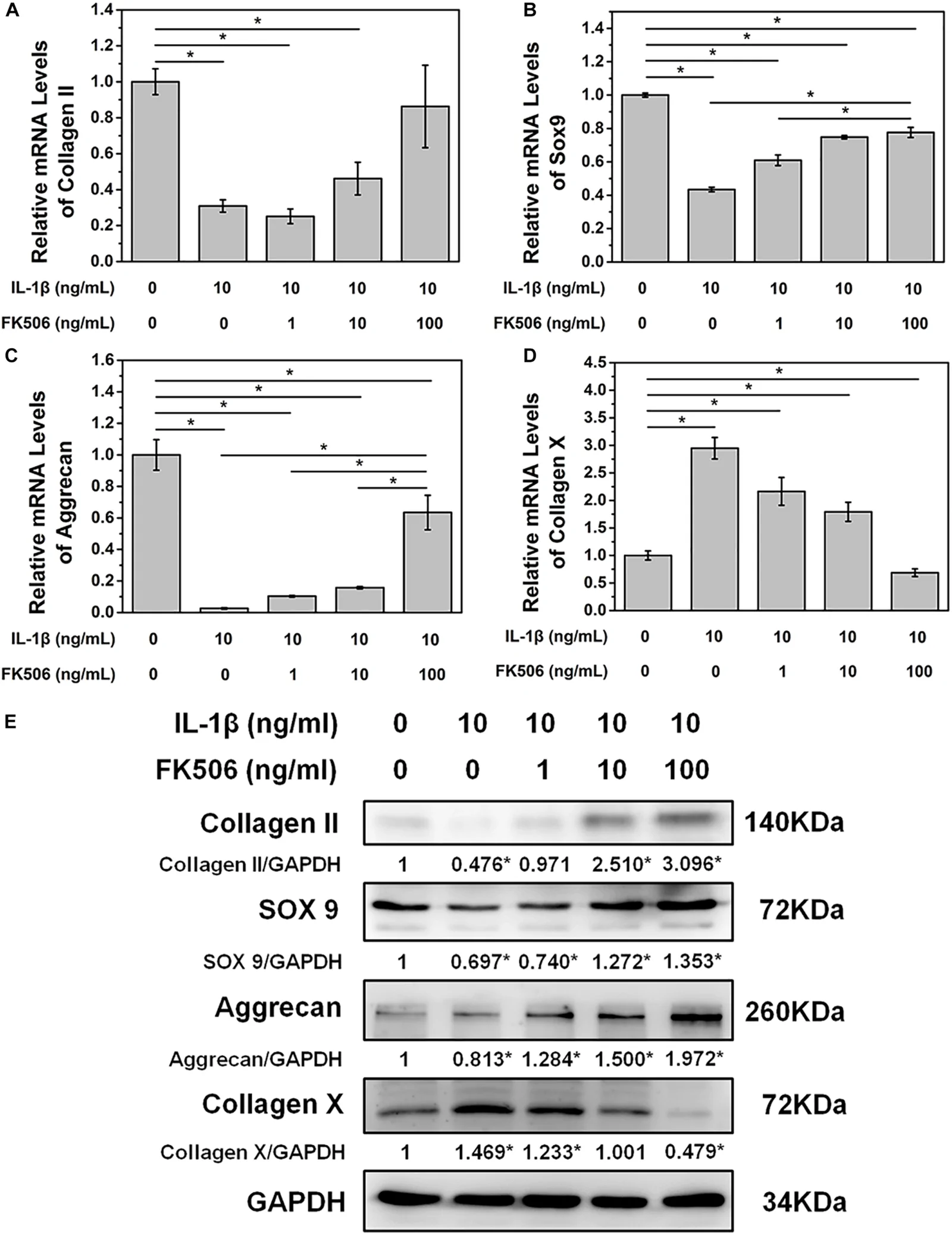
FK506 improves IL-1β-mediated NPC degeneration. **(A–C)** FK506 treatment can effectively alleviate the inhibition of IL-1β on the mRNA levels of type II collagen, aggrecan, and Sox9 (*p* < 0.05). These effects become more obvious as the level of FK506 increased. **(D)** FK506 treatment can effectively inhibit the stimulation of IL-1β on collagen X (*p* < 0.05), and this effect becomes more obvious as the concentration of FK506 increases. **(E)** FKk506 treatment can effectively alleviate the inhibition of IL-1β on the protein levels of type II collagen, aggrecan, and Sox9 and the stimulation of IL-1β on the protein levels collagen X (*p < 0.05).

To further demonstrate the effect of FK506 on NPCs, degeneration of NPCs was induced using interleukin-1 beta (IL-1β). Treatment doses higher than 10 ng/ml of IL-1β for 24 h sharply decreased the mRNA level of collagen type II. The concentration of 1 ng/ml of IL-1β could significantly increase the mRNA level of collagen type II, which may be caused by cellular stress response as we speculated (Figure 1B). Subsequently, we evaluated the NP gene and protein expression levels of collagen type II, transcription factor SOX9, and aggrecan, which are favorable factors for NPCs, and collagen type X, an important expression factor for NP degeneration. The qRT-PCR results indicated that the expression level of collagen type II, SOX9, and aggrecan was significantly decreased in IL-1β-treated NPCs, while the expression level of collagen type X was increased. These findings indicated that degeneration of the NPCs had occurred (Figures 2A–D). Western blot results revealed that FK506 increased the levels of collagen type II, SOX9, and aggrecan and reduced the level of collagen type X in a dose-dependent manner compared to observations in the controls. Interestingly, in response to high-dose FK506 treatment, the protein expression levels of collagen type II and aggrecan were even higher than those of the control group, suggesting that FK506 could not only delay the progression of IL-1β-induced NP degeneration but could even reverse the process and further improve the function of NPCs under higher concentrations (Figure 2E).

### FK506 Treatment, and Not Calcineurin Inhibition, Increased SMAD2/3 Activation

**FIGURE 3:**
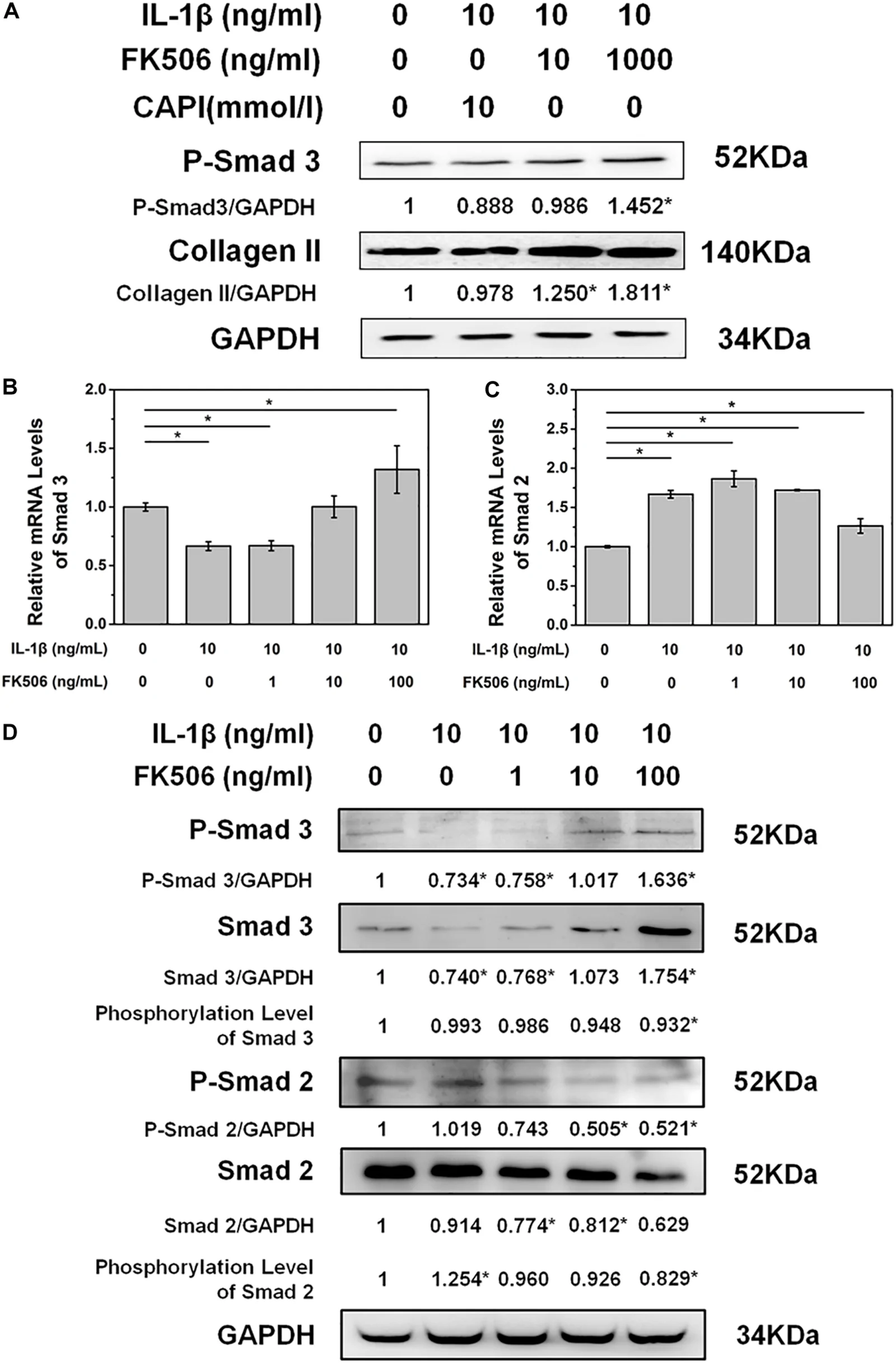
FK506 exerts its functions through the TGFβ pathway instead of the classical calcineurin inhibition pathway. **(A)** CAPI, a calcineurin inhibitor, failed to reverse the inhibition of IL-1β on type II collagen mRNA expression. Additionally, Smad3 was not phosphorylated. **(B,C)** IL1β treatment resulted in an increase in the mRNA level of Smad2 and a decrease in the mRNA level of Smad3. FK506 treatment can reverse this trend. **(D)** FK506 treatment can increase the protein level of Smad3 and increase the phosphorylation level of Smad3. In contrast, this treatment can reduce the protein level of Smad2 and reduce the phosphorylation level of Smad2 (*p < 0.05).

To further explore the mechanisms of FK506 in delaying IL-1β-induced NP degeneration, we explored the phosphorylation of key proteins in NPCs after FK506 treatment. As FK506 exerts an inhibitory effect on calcineurin, we first investigated the inhibition of the calcineurin pathway. Following IL-1β-induced NPC degeneration, we treated cells with the simple calcineurin inhibitor CAPI. However, the expression level of collagen type II in CAPI-treated cells was not significantly different from that in the IL-1β-treated group, suggesting that calcineurin inhibition does not delay IVD degeneration and that FK506 delays IVD degeneration through mechanisms other than the calcineurin pathway. Additionally, CAPI did not affect the phosphorylation level of SMAD 3, a critical protein within the TGF-β signaling pathway (Figure 3A). In contrast, FK506 treatment increased the phosphorylation levels of SMAD 3. Therefore, we further explored the relationship between FK506 and the TGF-β signaling pathway, and we found that the mRNA expression level of SMAD 3 was increased in NPCs after FK506 treatment, indicating that FK506 can delay NP degeneration through the TGF-β pathway (Figure 3B). We also observed that FK506 exerts an inhibitory effect on the mRNA expression of SMAD2 (Figure 3C). Further studies demonstrated that FK506 could activate the phosphorylation of Smad 3 in a dose-dependent manner while inhibiting both the protein and phosphorylation levels of Smad 2 (Figure 3D). In summary, FK506 exerts its function of delaying NP degeneration by activating the key protein Smad 3 to further activate TGF-β pathway.

### Inhibition of TGF-β Receptor Activation Weakens the Protective Effect of FK506 on IVD Degeneration

**FIGURE 4:**
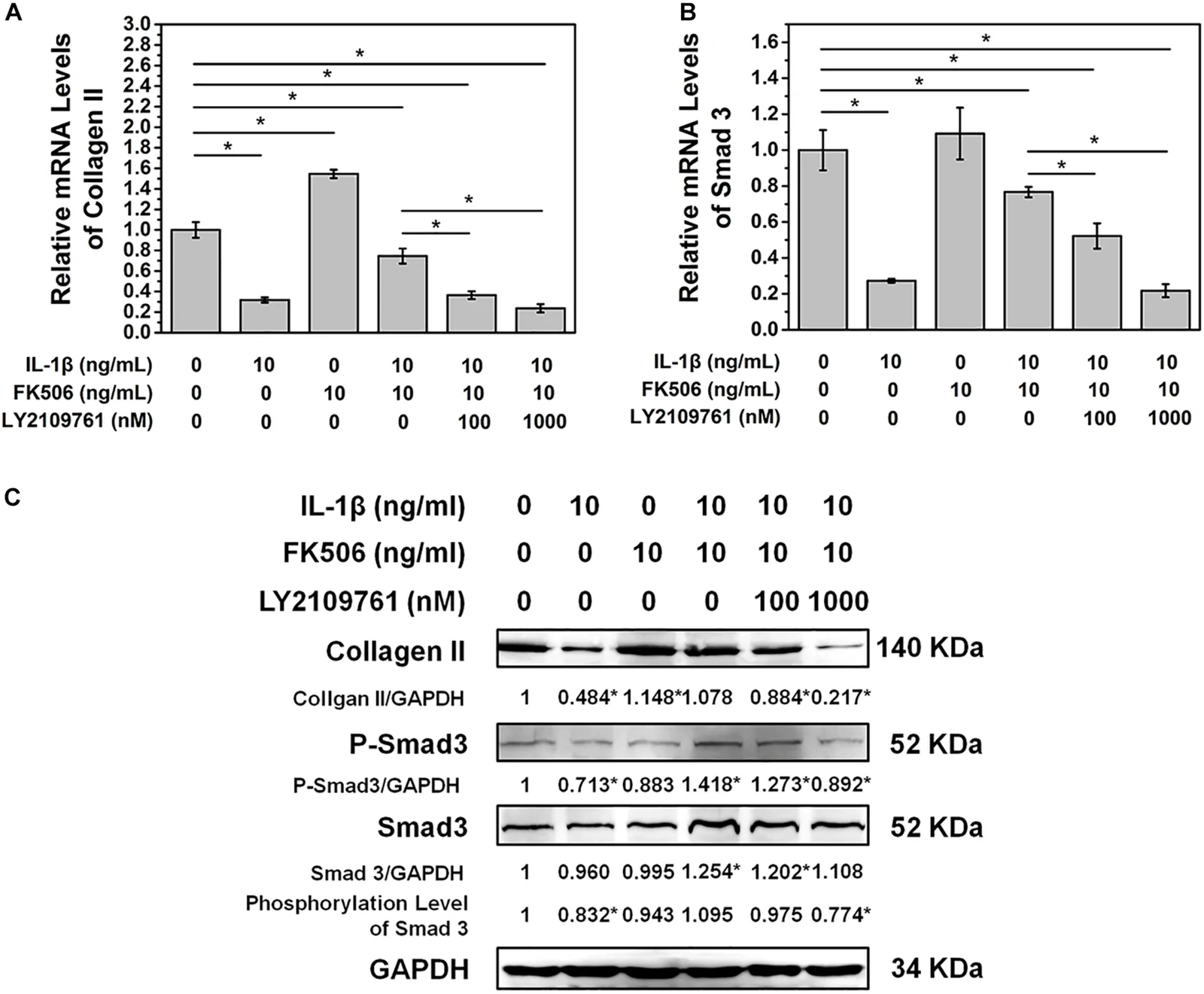
FK506 exerts its function through the TGFβ pathway. After treatment with LY2109761, a TGF β pathway inhibitor **(A,B)**. The protective effect of FK506 on the matrix components of NPCs was decreased, and the mRNA level of Smad3 and type II collagen was decreased. **(C)** The protein levels of type II collagen and Smad3 were reduced, and the phosphorylation level of Smad3 was also reduced (*p < 0.05).

To further confirm that FK506 functions through the TGF-β pathway, TGF-β inhibitor LY2109761 was added to the cultures of NP cells with FK506. LY2109761 specifically inhibited the TGF-β pathway in a dose-dependent manner, as evidenced by a decrease in the mRNA and phosphorylation level of Smad 3 as the concentration of LY2109761 increased (Figures 4A,C). Additionally, the gene and protein expression levels of collagen type II decreased after LY2109761 treatment (Figures 4B,C), suggesting that LY2109761 blocked the ability of FK506 to delay NP degeneration. The above results demonstrated that FK506 does exert its function of inhibiting IVD degeneration through the TGF-β pathway.

### Animal Experiment Results

**FIGURE 5:**
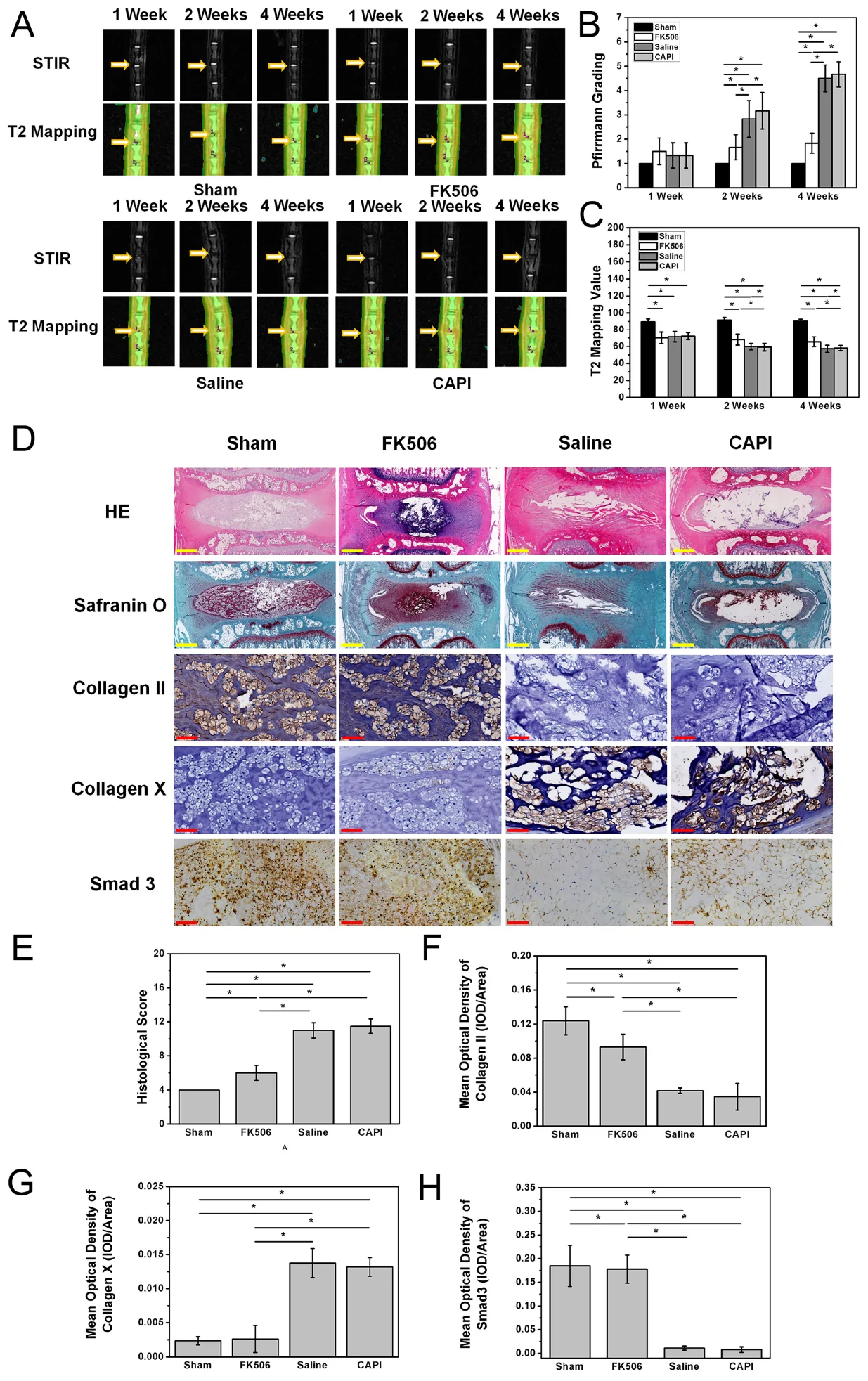
FK506 could relieve the IVD degeneration *in vivo*. **(A)** Magnetic resonance imaging findings after FK506 injection in rat caudal intervertebral disks, T2 signal intensity was stronger in the FK506-injected disks than that in control disks and CAPI group, but lower than that in Sham group. **(B)** Pfirrmann grading was significant lower on the 4th week of FK506 compared with control disks and CAPI group. **(C)** T2 value showed a significant better result of FK506. **(D)** Histological analysis of the intervertebral disks by hematoxylin and eosin staining revealed that many NPCs could be observed in the FK506 group, while NPCs were replaced by fibrocartilaginous tissue in the control groups and the CAPI group. Type II collagen immunohistochemical staining indicated that more Collagen II could be detected in the FK506 group at the 4th week, while Collagen X exhibited the opposite trend. Immunohistochemical staining revealed that Smad3 expression was elevated in the FK506 group at the 4th week. **(E)** Quantification of Collagen II immunohistochemical staining demonstrated that the FK506 group possessed a significantly higher mean optical density compared to that of the other groups (*p < 0.05). **(F)** Quantification of Collagen X immunohistochemical staining indicated that the FK506 group exhibited a significantly lower mean optical density compared to that of the other groups (*p < 0.05). **(G, H)** Quantification of Smad3 immunohistochemical staining revealed that the FK506 group possessed a significantly higher mean optical density compared to that of the control groups (*p < 0.05). Yellow scale bar = 500 μm; Red scale bar = 50 μm.

MRI results from the animal model showed a slight decrease in the T2 phase signal in all groups, indicating a successful animal model. MRI results of the second week revealed a decrease of the T2 phase signal in the saline-treated group and the CAPI-treated group, while a high signal was observed in the FK506-treated group. MRI results from the fourth week showed a more significant difference where the T2 phase signal in the intervertebral disk was further decreased in the saline-treated group and the CAPI-treated group and exhibited a morphology that included dark disks and a further reduced intervertebral space. In contrast, the FK506-treated group showed no difference in the T2 phase signal compared to that from the previous 2 weeks, and this group also possessed a normal intervertebral space (Figure 5A). Quantitative analysis revealed a lower Pfirrmann grade for the FK506-treated group compared to that of the saline-treated and the CAPI-treated group from the second week, and this difference was significant by the fourth week (Figure 5B). Furthermore, T2 value assessed by T2 mapping also showed a better result for FK506 (Figure 5C).

Histological staining of the 4-week samples showed that the volume of the intervertebral disk nucleus in the saline-treated and CAPI-treated groups was reduced or that the nucleus had even disappeared and that the structure of annulus fibrosus was disordered. The boundary between the annulus fibrosus and the NP became unclear. Some of the NP tissues were replaced by fibrous tissue, while others were damaged or fibrotic. In the FK506-treated group, the boundary along the surrounding annulus fibrosis was clear and intact and possessed a good morphology. The boundary between the NP and the annulus fibrosus remained clear. The cells in the NP tissues were significantly increased compared to those in the saline-treated and the CAPI-treated groups. The annulus fibrosus possessed a regular ring structure with neatly arranged layers and showed no significant degradation. More importantly, the intervertebral space in the FK506-treated group was significantly larger than that of the saline-treated and the CAPI-treated group (Figure 5D). We further quantitatively analyzed the morphology (Table 1), and although no difference in the morphological results was observed between the saline-treated and the CAPI-treated groups, a significant difference was observed when comparing these groups to the FK506-treated group (Figure 5E). The above results indicate that simple calcineurin treatment does not delay IVD degeneration, while FK506 can delay or even reverse the degeneration of intervertebral disk nucleus cells.

Finally, specific immunostaining for collagen type II, collagen type X, and SMAD 3 in NPCs was performed (Figure 5D). Immunohistochemistry results revealed that the collagen type II content in the FK506-treated group was much higher than that in the saline group and CAPI-treated group at the second and fourth week and did not exhibit significant differences compared to that in the saline-treated group (Figure 5F). Additionally, the expression level of collagen type X significantly was decreased compared to that of the other two groups (Figure 5G). The above results confirm the protective effect of FK506 on IVD *in vivo*. Additionally, the expression level of SMAD 3 in the FK506-treated group was also higher than that in the other two groups, suggesting that FK506 exerts its function through the TGF-β pathway (Figure 5H).

## Discussion

Intervertebral disk degeneration is a complex process with many factors underlying its pathophysiological mechanisms, and these include loss of proteoglycan, water, and type II collagen (Yoon and Patel, 2006), genetic inheritance, age, and insufficient transport of metabolites (Adams and Roughley, 2006). The inflammatory response accompanied by IVD degeneration has become a topic of interest for researchers, and inhibition of the inflammatory response has become a key strategy for delaying or even reversing the degeneration of the intervertebral disk. Excessive inflammatory mediators and factors can cause an imbalance between the synthesis and metabolism in NP extracellular matrix and can reduce the extracellular matrix, cell viability, and hydration capacity in NP, ultimately causing an inability to maintain the tension in the intervertebral disk and eventually IVD degeneration (Freemont, 2009). As an effective immunosuppressive agent, FK506 can inhibit immune-inflammatory responses. We hypothesized that FK506 can delay the degeneration of NPCs by reducing the inflammatory response in NPCs. Large amounts of IL-1β are often present in the degenerative intervertebral disks, and the accumulation of this factor leads to degeneration of the intervertebral disk. Previous studies have shown that treatment of human disk cells with IL-1 induces an imbalance between catabolic and anabolic events, and these are responses that represent the changes observed during disk degeneration (Maitre et al., 2005). We simulated the degeneration process in NPCs under the stimulation of inflammatory response by adding exogenous IL-1β. We then treated IL-1β pretreated NPCs with FK506, and the results revealed that FK506 could increase the number of NPCs and improve cell function.

Type II collagen (Kluba et al., 2005; Adams and Roughley, 2006; Melrose et al., 2008; Chan et al., 2011; Hayes et al., 2011; Ciapetti et al., 2012), Aggrecan and SOX9 (Sive et al., 2002) in the NPCs are closely related to the severity of disk degeneration. When the intervertebral disk degenerates, the content of proteoglycan and collagen type II within the extracellular matrix is decreased. Besides, when the surrounding biochemical conditions change, the NPCs lose their normal phenotype and transform into hypertrophic cells in which collagen type X replaces collagen type II (Gan et al., 2003). Our *in vitro* studies demonstrated that FK506 significantly increased the gene and protein expression levels of type II collagen, aggrecan, and SOX 9 in the intervertebral disk matrix while inhibiting the expression of the IVD degeneration indicator collagen type X.

We further explored the specific mechanism of FK506 in protecting the intervertebral disk. The protective effect of FK506 on cartilage is conclusive. The controversial effect of the Cn/NFAT pathway on cartilage indicates that it is unlikely to play a decisive role in the protective effect of FK506 on cartilage. Based on the protective effect of FK506 on NPCs, we first treated degenerative NPCs with a simple calcineurin inhibitor (CAPI); however, CAPI did not delay the degeneration of NPCs, and the expression level of type II collagen was equivalent to that of the IL-1β-induced degeneration group.

Rats were selected as the research objects in this work due to ease of use in simple and reproducible procedures, and availability of relevant antibodies (Qian et al., 2019). Besides, rat caudal intervertebral disks have similarity to human biochemical components (Borde et al., 2015). Rat caudal disks have been widely used in many studies, especially in morphologic, biologic, and molecular research (Grunert et al., 2014; Ji et al., 2018; Qian et al., 2019). Our animal experiments further confirmed that simple calcineurin inhibitors could not delay the degeneration of the intervertebral disk, as they yielded imaging and histological staining results comparable to those of the intervertebral disk degeneration group. The above results suggest that FK506 does not exert its function of delaying the degeneration of NPCs by inhibiting calcineurin.

With the continuous development of scientific research, studies have found that TGF-β exerts an inhibitory effect on the immunoinflammatory response. By influencing the development of immune cells and their differentiation, tolerance induction, and homeostasis, TGF-β plays a vital role in the treatment of many diseases (Sheng et al., 2015), including angiogenesis (Massagué et al., 2000) and wound healing (Ashraf et al., 2009; Wang et al., 2011). TGF-β exerts its functions mainly via Foxp3-dependent and independent mechanisms (Yoshimura and Muto, 2010). TGF-β transduces signals by binding to type I and type II (TβR-I and TβR-II) membrane receptors, and activated receptors exert their function by specifically recognizing and phosphorylating Smad signaling intermediates. Smad 2 and Smad 3 play essential roles in Foxp3 induction and cytokine suppression (Takimoto et al., 2010).

Interestingly, our results confirmed that FK506 could decrease the phosphorylation level of Smad2 and reduce the gene expression. However, FK506 can also increase the phosphorylation level and gene expression of Smad3. Additionally, animal experiments demonstrated that Smad 3 is highly expressed in the nucleus of NPCs after FK506 treatment. Smad2 and Smad3 possess highly homogeneous amino acid sequences. Smad2 possesses two more amino acid fragments than does Smad3, and these additions to the TID fragment enable Smad3 to bind directly to DNA (Rissoan et al., 1999). This leads to a variety of function differences between Smad2 and Smad3. Smad3 plays an important role in inhibiting T-cell activation and proliferation through the TGFβ pathway (Letterio, 2005), while Smad2 does not. A series of *in vivo* and *in vitro* studies have found that the incidence of inflammation in Smad 3-deficient patients is much higher than that in normal patients, suggesting that Smad 3 is important for anti-inflammation. Roberts et al. (2010) even proposed that Smad 3 is a key player in TGF-β-dependent pathogenetic mechanisms. The above results indicate that FK506 does exert its function of delaying the degeneration of NPCs by activating Smad 3 through the TGF-β pathway.

Although we did not study the specific mechanism by which FK506 regulates TGF-β, we speculate that FK506 regulates the TGF-β pathway via FK506 binding-protein 12 (FKBP12). FKBP12, an abundant immunophilin, is a physiologic regulator of cell cycle activity and is a key protein in the initiation of the cell cycle (Aghdasi et al., 2001). A small number of animal experiments have demonstrated that FKBP12 knock-out can cause problems such as heart defects, cell cycle arrest, and even death. Unfortunately, this key protein inhibits TGF-β-related downstream pathways by inhibiting TβR-I phosphorylation by TβR-II (Chen et al., 2014). When the homeostasis of this pathway is disrupted and FKBP12 is overexpressed, it inhibits the TGF-β pathway to cause degeneration of the intervertebral disk. Furthermore, we speculated that p-Smad3 may further regulate the expression of downstream NFκB, thereby reducing the impact of the inflammatory response, based on our former research (Yang et al., 2015, 2016). Based on the above speculation, we aim to initiate further research studies regarding this mechanism.

Although we have revealed the protective role of FK506 in the IVD degeneration, there are still some shortcomings in our work. On one hand, the observation time in animal experiments is short, and we will extend the observation time further in future. On the other hand, the current work did not make *in vivo* histological evaluation at different time points. It makes the histological evaluation results not intuitive enough.

Above all, this study demonstrated the positive effect of FK506 on delaying the degeneration of the intervertebral disk. Importantly, we also found that FK506 does not act on NPCs through inhibiting calmodulin. This study further expanded the knowledge of the biological function of FK506 and provides novel ideas for the clinical treatment and prevention of IVD degeneration. In the future, FK506 injection could be used as a therapy to delay intervertebral disk degeneration.

## Data Availability Statement

The original contributions presented in the study are included in the article/supplementary material, further inquiries can be directed to the corresponding author/s.

## Ethics Statement

The animal study was reviewed and approved by Ethics Committee of The First Affiliated Hospital of Soochow University. All the experimental procedures were carried out in strict accordance with Declaration of Helsinki (1964) and the Laboratory Animal Guidelines for Ethical Review of Animal Welfare (GB/T 35892-2018, China).

## Author Contributions

JG, YW, QY, CW, and HY performed the experiments and collected the data. JG analyzed the data, assembled the figures, and wrote the manuscript. JG and JZ interpreted the data. YW, QY, CW, and HY searched the literature. HY and JZ designed the studies and collected the funds. All the authors read and approved the final manuscript.

## Correction Note

A correction has been made to this article. Details can be found at: 10.3389/fcell.2026.1989743.

## Conflict of Interest

The authors declare that the research was conducted in the absence of any commercial or financial relationships that could be construed as a potential conflict of interest.
