# Correction: FK506 induces the TGF-β1/Smad3 pathway independently of calcineurin inhibition to prevent intervertebral disk degeneration

**DOI:** 10.3389/fcell.2026.1989743

**Published:** 2026-10-20

**Authors:** Jun Ge, Yingjie Wang, Qi Yan, Cenhao Wu, Hao Yu, Huilin Yang, Jun Zou

**Affiliations:** Department of Orthopaedic Surgery, The First Affiliated Hospital of Soochow University, Suzhou, China

**Keywords:** FK506, disk degeneration, TGFβ, Smad3, signal pathway

There was a mistake in Figure 5 as published. An incorrect MRI image was inadvertently used for the CAPI group at the 1-week time point in Figure 5A during figure assembly. The corrected Figure 5 appears below.

**FIGURE 5:**
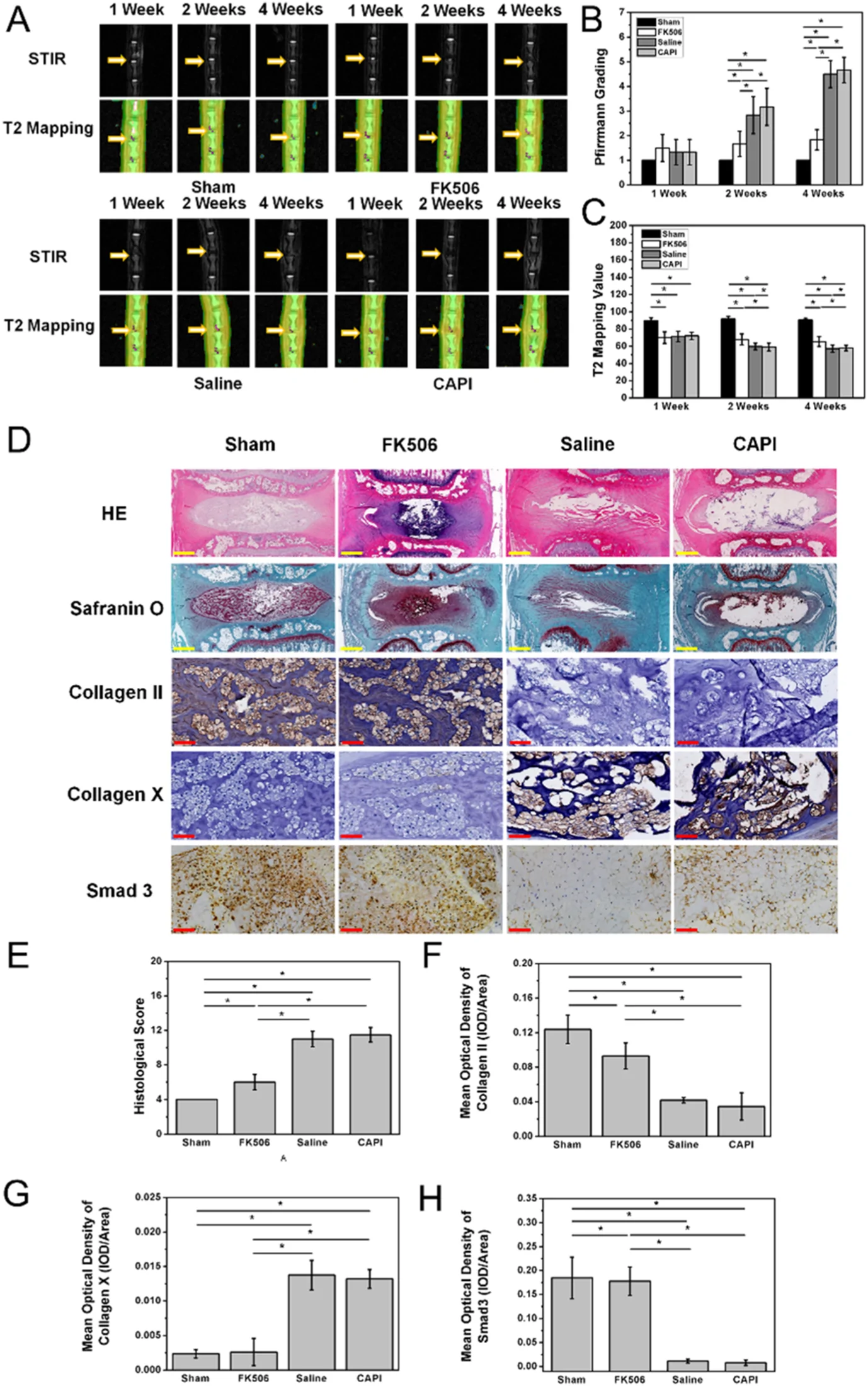
FK506 could relieve the IVD degeneration *in vivo*. **(A)** Magnetic resonance imaging findings after FK506 injection in rat caudal intervertebral disks, T2 signal intensity was stronger in the FK506-injected disks than that in control disks and CAPI group, but lower than that in Sham group. **(B)** Pfirrmann grading was significant lower on the 4th week of FK506 compared with control disks and CAPI group. **(C)** T2 value showed a significant better result of FK506. **(D)** Histological analysis of the intervertebral disks by hematoxylin and eosin staining revealed that many NPCs could be observed in the FK506 group, while NPCs were replaced by fibrocartilaginous tissue in the control groups and the CAPI group. Type II collagen immunohistochemical staining indicated that more Collagen II could be detected in the FK506 group at the 4th week, while Collagen X exhibited the opposite trend. Immunohistochemical staining revealed that Smad3 expression was elevated in the FK506 group at the 4th week. **(E)** Quantification of Collagen II immunohistochemical staining demonstrated that the FK506 group possessed a significantly higher mean optical density compared to that of the other groups (*p < 0.05). **(F)** Quantification of Collagen X immunohistochemical staining indicated that the FK506 group exhibited a significantly lower mean optical density compared to that of the other groups (*p < 0.05). **(G)** Quantification of Smad3 immunohistochemical staining revealed that the FK506 group possessed a significantly higher mean optical density compared to that of the control groups (*p < 0.05). Yellow scale bar = 500 μm; Red scale bar = 50 μm.

The original version of this article has been updated.

